# Dual-Gendered Leadership: Gender-Inclusive Scientific-Political Public Health Communication Supporting Government COVID-19 Responses in Atlantic Canada

**DOI:** 10.3390/healthcare9101345

**Published:** 2021-10-10

**Authors:** Haorui Wu, Jason Mackenzie

**Affiliations:** 1 School of Social Work, Dalhousie University, Halifax, NS B3H 4R2, Canada; js891323@dal.ca; 2 Natural Hazards Center, University of Colorado Boulder, Boulder, CO 80309, USA

**Keywords:** COVID-19 emergency responses, public health interventions, dual-gendered leadership, women chief medical officers, men prime minister and premiers, Atlantic Canada, scientific-political communication, media coverage

## Abstract

This research aims to identify the influence of woman leadership on improving the traditional man-dominated scientific-political communication towards positive COVID-19-driven public health interventions. Across Canada, dual-gendered leadership (women chief medical officers and men prime minister/premiers) at both federal and provincial levels illustrated a positive approach to “flatten the curve” during the first and second waves of COVID-19. With the four provinces of New Brunswick, Newfoundland and Labrador, Nova Scotia, and Prince Edward Island, Atlantic Canada formed the “Atlantic Bubble”, which has become a great example domestically and internationally of successfully mitigating the pandemic while maintaining societal operation. Three provinces have benefitted from this complementary dual-gendered leadership. This case study utilized a scoping media coverage review approach, quantitatively examining how gender-inclusive scientific-political cooperation supported effective provincial responses in Atlantic Canada during the first two waves of COVID-19. This case study discovers that (1) at the provincial government level, woman leadership of mitigation, advocating, and coordination encouraged provincial authorities to adapt science-based interventions and deliver consistent and supportive public health information to the general public; and (2) at the community level, this dual-gendered leadership advanced community cohesion toward managing the community-based spread of COVID-19. Future studies may apply a longitudinal, retrospective approach with Canada-wide or cross-national comparison to further evaluate the strengths and weaknesses of dual-gendered leadership.

## 1. Introduction

Gender-specific themes have attracted attention in the field of disaster and emergency management. Previous research has highlighted various women’s contributions within an entire lifecycle of an extreme event [1,2] and across different types of disaster events [3,4]. Within the emergency response stage, although scholars have significantly contributed to gender-driven vulnerabilities and gender-specific contributions [5,6], less attention has been paid to gender differences that take place in the process of emergency decision making. Possible reasons are grounded in the historical context of man-dominated disaster and emergency management leadership [7]. With an increasing number of women assuming political leadership, this research deficit has potentially been a factor jeopardizing the advancement of an inclusive approach for disaster risk reduction and sustainable community development [8].

In the current COVID-19 climate, public media have portrayed the leadership excellence of women political leaders worldwide, such as Jacinda Ardern (New Zealand’s Prime Minister) and Angela Merkel (Germany’s Chancellor), as superior to their counterparts who are men [9]. The achievements of women political leaders have featured swift responses to the pandemic and effective communication strategies that unite their citizenry to adhere to public health protocols worldwide [10]. It is critical to note that this public health crisis also highlighted the leadership role of chief medical doctors/advisors, who provide science-based evidence to support the public health decision making of political leaders [11]. Although research has recognized the women chief medical doctors’ contributions [12], the influence of their roles combining both science and political obligations on scientific-political communication remains inadequate, especially in terms of their collaboration with men political leaders to customize community-driven public health interventions.

Across Canada, unique, dual-gendered leadership, namely, a woman chief-medical-officer (CMO) and a man prime minister (PM)/premier at both federal and provincial levels, was established before the COVID-19 pandemic. This leadership had swiftly responded to the current public health emergency and successfully “flattened the curve” during the first and second waves of COVID-19 [13]. This scientific-political leadership always presented together in official media releases, informing the general public of the newest public health interventions. Among the ten provinces and three territories across Canada (and the federal government of Canada), all fourteen federal and provincial governments are led by men. Correspondingly, among the fourteen federal and provincial CMOs, however, 50% of them (seven) are women (including Canada’s chief medical officer, Dr. Theresa Tam) [14]. Furthermore, there are also women in public health officer positions in the major metropolitan areas across the country (e.g., Vancouver, Toronto, and Ottawa) [15]. By the first anniversary of COVID-19 in Canada, Atlantic Canada (New Brunswick, Newfoundland and Labrador, Nova Scotia, and Prince Edward Island, known as the “Atlantic Bubble”) became a textbook example of success in the mitigation of the pandemic, both domestically and internationally [16]. Three out of these four provinces featured this complementary dual-gendered leadership (see Table 1). This dual-gendered leadership demonstrates the effectiveness of translating complex information of an unprecedented infectious threat and science-based public health interventions into easily understandable language through daily press conferences and media briefings across their provinces. Based on the phenomenon of dual-gendered leadership, this case study aims to identify the influence of woman leadership on improving the traditional man-dominated scientific-political communication towards positive COVID-19-driven public health interventions. This article adheres to the Government of Canada’s guidelines regarding the use of gender and sex with respect to research [17]. As such, the socially constructed titles (gender) of man and woman are utilized in this paper rather than the biological attributes (sex) of male and female when referencing the dual-gendered leadership roles within the political and public health fields.

## 2. Women Leaders

Although women have continually been underrepresented in political and professional leadership positions [19,20], as indicated above, woman leadership has been recognized in the current public health emergency in the global and Canadian settings. Hence, COVID-19 has generated a valuable opportunity to thoroughly investigate gender-inclusive leadership in the man-dominated disaster and emergency management domain. This literature review briefly portrays the landscape of woman leadership during emergency response.

### 2.1. Women’s Leadership in Political and Professional Realms

Extreme events caused by natural hazards (e.g., hurricanes and pandemics), technical accidents (e.g., traffic accidents and nuclear pollution), and intentional (e.g., civil wars and terrorist attacks) build a platform to explore the various capacities of political leaders [21]. Historically, man-dominant leadership has been mainstreamed in the field of disaster and emergency management [22]. Aggressive leaders, generally stereotyped as men, have been preferred during emergency response immediately after disaster because of the masculine protectionism toward citizens, especially after willful violence [23]. The general attitudes towards masculine protectionism might reinforce men taking leadership. However, the diverse requirements of disaster survivors revolve around physical protection and engage social, cultural, and economic dimensions, which undermine this man-dominant leadership [22]. Woman leadership has gradually been transforming this landscape by focusing on overall disaster mitigation [24]. In particular, compared with the man-only leadership, Funk [25] indicates that women mayors in the U.S. released disaster mitigation policies to prevent local risks and reduce negative social and economic consequences prior to the declaration of public emergency of COVID-19. Furthermore, the increasing number of women leaders not only benefit citizens affected by disaster, but also influence their nation’s bilateral foreign aid to other international communities regarding disaster risk reduction [26]. Hicks and colleagues [27] indicate that the higher the share of women in the national leadership corps, the more foreign aid the country would provide to support their international peers in the wake of a disaster.

In the field of medicine and healthcare, women represent seven out of ten healthcare professionals [28] and 90% of all nurses internationally [29]; however, women are underrepresented in the most prestigious leadership roles (e.g., the heads of the healthcare service organizations) [30]. As an illustration, in Canada, although women comprise 41% of all physicians and 63% of all medical students, there are only two (12%) serving as deans among 17 faculties of medicine at universities across Canada [31]. Furthermore, although approximately 70% of Canadian health and social care employees are women, they have been historically underrepresented in leadership roles (e.g., the heads of hospitals and departments) [32]. Promoting women’s leadership roles not only influences high-level interventions advocating for gender equality, but also presents role models and educates the next generation of women leaders [33]. Fitzpatrick [34], for example, asserts that women Canadian CMOs’ leadership during the COVID-19 pandemic became role models to their women colleagues and students in the fields of healthcare and social service. Similarly, the performance of these women leaders won the general public’s trust and gratitude and defied the stereotypical view that the general public only saw men in prestigious leadership positions [35].

Women are always disproportionately affected in the global context of climate change, disasters, and other crises [36]. Accordingly, merging both political and professional responsibilities in the current pandemic, dissimilarities between women’s and men’s political and professional preferences may result in divergent public health strategies and outcomes [37]. For instance, Bauer and colleagues [38] discovered that women leaders lean towards swift actions, avoiding potential risks according to model-based forecasting. Although public health decision making is a multidisciplinary and multi-engagement process, current research seems to isolate the women’s leadership achievements from the leadership corps at different levels of government. Analysis of both women’s political and professional responsibilities shows a paucity of research of women leaders and their collaborative performance with men political counterparts. This research deficit has caused a lack of deep discernment of the gender leadership gap. The narrowing of this gap would promote a gender-inclusive approach to improve the current and potential epidemiological hazards.

### 2.2. Gender-Inclusive Scientific-Political Communication during the COVID-19 Pandemic

Scientific-political communication, which bridges the scientific fact- and reality-driven governmental interventions, as well as the general public’s understanding and perception of reality [39], is extremely vital in response to the COVID-19 pandemic. Widely reported cases worldwide, in particular, have demonstrated that politicians could neglect healthcare professionals’ and scientists’ recommendations, putting citizens’ lives at risk [40]. Instead, the general public’s unsupportive behaviors toward the public health restrictions, such as anti-mask protests [41] and disobedience of social distancing and quarantine requirements [42], have negatively impacted the physical health, mental wellness, and overall well-being of themselves and others. Improving scientific-political communication is one of the most effective solutions [43]. Research has recommended strengthening the collective and interconnected societal benefits for the general public centers in the effective scientific-political communication strategies related to epidemical disasters [44]. Furthermore, gender diversity in scientific-political communication benefits the whole population, significantly raising public awareness and improving public education [45]. Research has rarely, if ever, examined the influence of man–woman cooperation in the realm of scientific-political communication.

Moreover, since the general public’s exposure to scientific and political issues has always been indirect through mass on-/off-line media [46], media coverage has shaped various grassroots behaviors reflecting their understanding and perception of public health risks [47]. Media can either contribute to gender equity or strengthen the stereotypical gender inequality dependent on a journalistic slant [12]. In the U.S., through the results of a survey experience of citizens, Bauer and colleagues [38] argue that political leaders’ gender has not significantly influenced the general public’s policy compliance. Instead, in Canada, women CMOs’ daily briefs of the pandemic streamed through public media channels gained most of the public’s respect and collaboration as trustworthy, professional, and contrarily, bringing gender-related critiques, abuse, and even threats [48]. These women leaders demonstrated that their science-specific expertise transcends political and social stereotypes. Gender transformative policy can fundamentally build women’s leadership roles in scientific-political communication [28]. Exploring these roles in scientific-political communication at the leadership corps level will inform the public health decision making at the government level. The improved public health policies will generate a ripple effect that could advance the general public’s health and well-being.

The gender leadership gap is outstanding in the man-dominated political and professional fields [49]. Amid the unprecedented global pandemic of COVID-19, emerging research focusing on international women leaders has predominantly highlighted that their decision-making capacity, including responding actions and communication approaches, have been better than their men counterparts [25,37,38]. The Canadian dual-gendered leadership provides a valuable opportunity to redress gender disparities in scientific-political communication by investigating women’s collaborative performance with their men political counterparts to strengthen public health communication. Taking Atlantic Canada as an example, this case study is guided by the question: How could women’s leadership influence the traditionally man-dominated scientific-political communication and improve political interventions regarding COVID-19 in the Atlantic Bubble?

## 3. Dual-Gendered Leadership in the Atlantic Bubble—A Case Study

Previous research has illustrated that public media are valuable sources to examine emergency response at both government and grassroots levels [21,50]. The COVID-19 pandemic relevant public health protocols enabled the digital public media to become the primary information source for the general public, especially during the lockdown periods. Since March 2020, at the federal government level, Canadian PM Justin Trudeau and CMO Dr. Theresa Tam have collaborated and informed the general public regarding national interventions widely covered by digital public media [51]. The four provinces in the Atlantic Bubble have utilized the same premier–CMO partnership and have administrated daily provincial, regional, and national updates to inform residents through public media. The digital public media has been a platform for the general public to understand pandemic-related public health interventions at federal, provincial, and municipal levels. Drawing from a phenomenological lens, this qualitative study will focus on digital public media data to identify the influence of woman leadership on improving the traditional man-dominated scientific-political communication towards positive COVID-19-driven public health interventions in Atlantic Canada.

### 3.1. Settings

With the third wave of COVID-19 receding in Canada, the Atlantic Bubble is “the envy of Canada” [52] (para. 12) due to its social, cultural, economic, and population characteristics as well as geography, travel restrictions, and adherence to public health guidelines [53]. Geographically, the four Atlantic provinces are the easternmost provinces of Canada, surrounded by the Atlantic Ocean. When COVID-19-driven strict international border control was effective in March 2020, all the international flights and ferries connoted with the Atlantic region were canceled, and the flights connecting the Atlantic region with other parts of Canada were significantly redacted [54]. These features effectively reduced the imported COVID-19 cases into the Atlantic region.

Within the Atlantic Bubble, the Maritimes of the four provinces features diverse similar societal characteristics. For instance, Atlantic Canada presents a small population, a low urban density, and the highest share of rural residents in Canada [55]. Although Canada accepts tens of thousands of immigrants per year, in 2020, only 5% of the total new immigrants settled in the Atlantic region [56]. Hence, most residents in the Atlantic region have strong local ties. The economic development in the Atlantic region is even, with three out of four provinces ranked near the bottom of gross domestic product within the Canadian federation [57]. Since the political sector is strongly associated with other societal factors, the geographic, demographic, and economic similarities guarantee a homogeneous platform for a cross-provincial comparison within the region. These region-based similarities also reflect the significant societal differences, comparing the Atlantic region to other provinces. Hence, a cross-region comparison would not provide an accurate snapshot of other dual-gendered leadership due to the substantial societal differences.

### 3.2. Data Curation—A Scoping Public Media Coverage Review

The preferred reporting items for systematic reviews and meta-analyses (PRISMA) [58] was adapted to guide this scoping public media coverage review, including the following steps:

#### 3.2.1. Data Sources

According to Agarwal [59], the top two Canadian news websites are Canadian Broadcasting Corporation (CBC) News and CTV News, based on the authority, frequency, and scope of their media coverage. The Globe and Mail is the most widely read newspaper on weekdays and Saturdays in Canada [60]. During the pandemic, the Globe and Mail’s web-based news articles have virtually informed their loyal reader groups. These three public media have bilingual versions of English and French, reflecting both official languages in Canada. Since these four Atlantic provinces are predominantly English speaking, this study focuses on English news articles only.

#### 3.2.2. Search Keywords

The study developed the following two groups of keywords to establish the searching boundary. Group one was leadership corps, including the full name of premiers AND CMOs (see Table 1). Group two was created for terminologies involving COVID-19, including COVID-19, coronavirus, and variant. As an instance, the searching formula that was used for identifying news articles in New Brunswick (N.B.) is (Blaine Higgs AND Jennifer Russell) AND (COVID-19 OR coronavirus OR variant).

#### 3.2.3. Searching Period

Due to the time-sensitive and unpredicted characteristics of the current COVID-19 pandemic, this case study focuses on the first two waves of COVID-19 in order to initially explore the influence of dual-gendered leadership on provincial public health strategies in Atlantic Canada. The first two waves of COVID-19 in the Atlantic Region span from 23 January 2020 to 10 February 2021. On 23 January 2020, although Dr. Theresa Tam (CMO of Canada) initially declared that the risk of an outbreak of COVID-19 in Canada at the time was low, all the provinces and territories across Canada were on heightened alert [61]. On 10 February 2021, Dr. Robert Strang (CMO of N.S.) illustrated that the second wave had almost ended [62]. Since N.S. was the Atlantic epicenter during the first and second waves of COVID-19, the search ended on 10 February 2021.

#### 3.2.4. Searching Results

As shown in Table 2, the initial search yielded 2558 articles, including 1728 articles from the Atlantic region. The following two inclusion and exclusion criteria were established to further narrow initial search results. Criterion 1. Media have their perspectives to interpret the same news from different angles [63]. Commonly, the same news topic is covered by three outlets simultaneously (usually, these articles are available on the same day or within consecutive days). Where syndication occurred across media outlets was considered duplication and removed from the search. Criterion 2. This study concentrates on the influence of gender on scientific-political communication during the emergency response to the first two waves of COVID-19. Hence, news articles regarding COVID-19-related daily updates, such as the number of new infected cases, active cases, completed tests, and administrated doses of the vaccine, were not the main focus of this study. Daily updates that solely focused on active cases, completed tests, and administered doses were excluded from this research. These daily updates, however, assist the authors to portray a comprehensive landscape to assist in understanding other news articles. After applying these two criteria, the number of final reviewed news articles is 83, shown in Table 2. The complete list of reviewed articles, including their author(s), title, date of publication, and uniform resources locator (URL), is available on a data repository of DesignSafe-CI.org [64].

#### 3.2.5. Data Analysis

A content analysis approach, which is well known for its analytical and conceptual rigor and suited for deep examination of gender-inclusive scientific-political communication [65,66], was employed. Emerging theme strategies were used to compare and synthesize media articles from among the four Atlantic provinces. The news articles identified at the federal level served as background information to assist the authors to further elaborate on gender influence. Most of these articles consist of related news media and original data resources, especially those from the official government websites, where relevant policies and/or interventions were released. These extended resources provide a supplemental background that assisted the authors to deeply understand the public health communication within each province. Although the four provinces share societal similarities and present significant differences (including geographic, social, economic, and political), these characteristics were also embedded in the data analysis in order to develop both independent and collective themes and facilitate the cross-provincial, gender-based analysis in the Atlantic Bubble.

Both authors have completed professional training in social work and equipped social work-specific research capacities. They applied a phenomenological lens to explore the dual-gender leadership’s various influences. Built on an interpretivist paradigm [67], this study was grounded in the assumption that the dual-gender leadership in Atlantic Canada demonstrated more positive impacts on scientific-political communication than the traditional man-only leadership could during COVID-19. Accordingly, the two authors reviewed all the news articles and developed codes independently, then discussed and merged themes collaboratively through two-round data analysis (see Table 3) with deductive and inductive approaches [66]. The first-round indicative analysis enabled the authors to conduct cross-provincial comparison and identify gender-based strategies, which merge a comprehensive gender-informed perspective to examine and synthesize related data. Since three provinces (N.B., P.E.I., and N.L.) benefit from this dual-gendered leadership, while N.S. remains traditional man-only leadership, the cross-provincial comparison approach interpretively discoursed the pandemic-driven scientific-political communication and public health decision making within these provinces’ unique backgrounds. The indicative analysis enables the researchers to develop seven sub-categories, supported by various codes shown in Table 3.

The second-round data analysis applied a deductive approach, which stimulated the two authors to retrospectively integrate these seven sub-categories into three levels of major themes, indicating the dual-gender leadership’s influences on provincial governments’ public health decision making, dissemination of public health information, and local communities. These themes, reporting in the next section, establish a gender-based approach that contributed to a nuanced understanding of leadership influences within the scientific-political communication and relevant critical decision making by identifying emergent themes and their correlation with gender-specific policy leadership.

## 4. Findings

This section presents the benefits of dual-gendered leadership in the following three aspects: (1) the contributions of decision making at the federal and provincial government levels, (2) dissemination of public health information to the general public, and (3) the grassroots-level responses. Subcategories were supported by related news articles. This approach enabled the two-way strategy of examining the dual-gendered leadership’s commitment between government and residents. In order to clearly demonstrate the women CMO’s contribution in the dual-gendered leadership, the CMOs are referred by their professional title and last name (e.g., Dr. Tam and Dr. Russell). The men PM, premiers, and CMO are referred to by political title and last name (e.g., Canada PM, N.B. Premier, and N.S. CMO).

### 4.1. Benefits of Gender-Inclusive Leadership at the Provincial Government Level

Within the provincial leadership corps, women CMOs generally contributed to the provincial public health emergency response decision making in the three roles: mitigation, advocacy, and coordination.

#### 4.1.1. Mitigation Role: Giving Priority to Residents’ Health and Well-Being over Economic and Political Dimensions

The economic impacts of the pandemic, especially associated with mandatory business closures, play a critical role in governmental public health interventions [68]. The federal government of Canada followed a science-based reopening of the economy after the first lockdown [69]. Under this national policy, provinces and territories across Canada established their own processes. Although some provinces reported that their coping strategies have dramatically neglected their CMOs, medical, and other scientists’ input [70], leadership in the Atlantic region has successfully followed the scientific-driven approach, where gender may play a mitigating role.

Disaster and emergency management theories suggest that pandemic-driven emergency response should comprehensively mitigate various societal aspects, including economic, health, and social [71]. Among the Atlantic provinces, news releases indicate that N.S. with a man-only leadership exhibited a greater propensity to accept risk in response to the pandemic than neighboring provinces with dual-gendered leadership. As an illustration, after weeks of lockdown measures, the N.S. Premier stated, “We believe that we have found a balance between public safety and restarting our economy” [72] (para. 21). Throughout the first and second waves of the pandemic, N.S. leadership instituted protectionist measures to support economic stability at the expense of the varied needs of citizens.

In contrast, other provinces with dual-gendered leadership featured an approach that addressed the holistic needs of the residents through a protectionist approach rooted in risk mitigation. When faced with mounting pressure from business leaders in N.L., its premier, collaborating with Dr. Fitzgerald, referenced the economy and public health measures: “It’s not about picking one or the other. It’s about making sure we do all of it safely” [73] (para. 28) and “we will reopen the economy, but we must do it when it’s safe to do so and we must plan for it and be prepared if we have a [setback (sic)]” [74] (para. 11). These examples provide evidence that a comprehensive approach securing a balance between public health measures and the economy takes precedence in dual-gendered leadership. The women leaders appeared to prevent risks in advance due to the many unknowns associated with the rapidly evolving pandemic.

#### 4.1.2. Advocating Role: Focusing on Vulnerable and Stigmatized Populations

A comprehensive approach with respect to risk mitigation was also evident in dual-gendered leadership concerning vulnerable and stigmatized populations. This model in Atlantic Canada utilized messaging that supported these groups. Early data collected after the first wave of COVID-19 in Canada indicated that individuals in ethnocultural neighborhoods that were at a socioeconomic disadvantage, visible minorities living in crowded housing conditions, and those employed in occupations associated with greater risk of exposure to the virus face higher mortality rates than the general population [75]. Dr. Tam highlighted the risks posed by COVID-19 to Indigenous people due to health inequities, higher rates of underlying health conditions, and the challenge of communities located in remote areas. As such, the PM and Indigenous Services Canada responded with funding announcements and supported directed towards this population [76]. At the provincial level, the leadership crop released a clear message to support the essential workers who are not directly engaged with in-hospital COVID-19 patients (e.g., long-term care facility staff, food industry workers, and public transport sector employees) [77].

In Canada, people and communities of African descent have been experiencing various racism and discrimination [78]. Statistic Canada’s recent data show that COVID-19 illustrates these pre-existing inequities in Canadian society [79]. There were examples of stigmatization in the Atlantic provinces as a result of comments by senior leadership. N.S. Premier and CMO (man-only leadership) were criticized for singling out predominantly Black communities as “hotspots” for COVID-19 [80], while also admonishing youth for “living as COVID did not exist” [81] (para. 5). In N.B., after a health professional (African descent) did not self-isolate after traveling, they were labeled as “irresponsible” by N.B. Premier with threats of criminal charges being laid, despite the fact the investigation was not completed by health officials [82]. The premier was later identified on the Internet and faced discrimination and harassment from the public [83] before Dr. Russell encouraged people not to “stigmatize…ostracize or villainize” [84] (para. 25), showing compassion towards individuals who had tested positive for the virus. This evidence indicates that dual-gendered leadership placed specific emphasis on vulnerable and stigmatized populations during outbreaks of COVID-19 in the Atlantic region, reinforcing the collective needs of all citizens and taking particular actions to support the groups impacted disproportionately.

#### 4.1.3. Coordination Role: Improving the Cross-Departmental Collaboration

News articles consistently convey the significance of collaboration across jurisdictional boundaries, political affiliation, and across sectors as an effective method to support the development of policies that will address the needs of populations impacted by COVID-19. Cross-party collaboration occurred at the national level with the PM and Dr. Tam provided a technical briefing to “encourage cross-partisan unity” [85] (para. 4) on the subject of COVID-19 and updated modeling. In addition, this dual-gendered leadership held calls with the provincial counterparts providing support during the pandemic [86]. For instance, the PM raised the issue of using the Emergency Measures Act, which would offer sweeping powers to respond to the pandemic; however, a majority of the premiers rejected it for various reasons. Despite disagreement and political wrangling, it was noted that constant communication between Ottawa and the provinces was an effective method in the fight against COVID-19 [87].

As infected case numbers began to drop after lockdown measures in N.B., Dr. Russell noted this success was due to establishing a team of public health clinicians and participation in an inter-provincial task force providing timely updated information and sharing best practices [88]. Dr. Russell illustrated that the cross-party COVID-19 cabinet committee “has allowed us to have a really streamlined approach to decision-making which means there is a lot of unity in our messaging” [88] (para. 5). When preparing to ease lockdown restrictions in P.E.I., Dr. Morrison argued that no decisions would be made without consultation and “risk assessment with industry, government departments, businesses and communities” [89] (para. 5).

These cases assert that the dual-gendered leadership enhanced collaboration across jurisdictional, political, and sectoral boundaries and likely encouraged multiple voices to address policy decisions with a keen eye focused on public health. This has critical implications on streamlining and using effective messaging to a public bombarded with daily updates related to COVID-19.

### 4.2. Dissemination of Public Health Information to the General Public

With a high degree of emphasis placed on citizen engagement, consistent and supportive messaging must be provided in a manner that sparks trust and engages the public in risk communication, response, and mitigation [90]. Dual-gendered leadership features delivering consistent and supportive messaging to unite the residents.

#### 4.2.1. Delivering Consistent Messages, Avoiding Misdirecting Public Attention and Misinformation

The unpredictable characteristics of COVID-19 triggered leadership in each Atlantic province to provide inconsistent messaging at different points throughout the first and second waves. However, the provinces utilizing dual-gendered leadership have openly explained the lack of consistency regarding messaging with attempts to educate the public through various means.

N.S. leadership wavered on the use of face coverings initially and later recommended “residents to wear non-medical masks when physical distancing among strangers becomes difficult” [91] (para. 3). When faced with confusion among citizens of P.E.I., Dr. Morrison made an effort to clarify the difference between social isolation and social distancing and provide consistent messaging for travelers [92]. At the federal level, Dr. Tam and PM Trudeau referenced the spread of misinformation across the Internet and through social media. Dr. Tam cautioned citizens to be wary as information spreads “faster than the virus itself” [93] (para. 3).

Medical leadership utilized media outlets and personal messages to the public. Dr. Morrison regularly conducted weekly interviews with CBC News, providing direct information and messages to P.E.I. residents [94]. In N.B., there were multiple examples of Dr. Russell providing holiday tips [95], attending radio programs to answer listener questions [96,97], and recording videos to provide public health reminders in lieu of a daily briefing [98]. Despite these efforts, N.B. officials lamented the lack of success in messaging reaching young people, with Dr. Russell commenting, “I wish this would go viral… somebody do a TikTok on this” [99] (para. 14). Despite challenges reaching target groups and combating misinformation, there is evidence that the dual-gendered leadership uses direct communication and messaging to educate the public.

#### 4.2.2. Supportive Messaging to Build Trust with the General Public

Evidence shows that dual-gendered leadership utilized supportive and encouraging messages to the public in order to recognize successes and promote unity among individuals through public health measures. With the first presumptive case of infection in N.L., Dr. Fitzgerald encouraged citizens to self-monitor their mental health status [96]. Upon the easing of restrictions during the first wave, Dr. Russell cautioned citizens to maintain vigilance while the N.B. Premier praised their residents’ efforts [97,100].

Dr. Morrison exhibited supportive communication to P.E.I. inhabitants:

*“We’ve done so well and come so far… it’s my personal plea, and shared by the team: Now is the time for us to really stick with it and follow these measures for a bit longer, and now is not the time to throw caution to the wind.”*

[101] (para. 8)

The man-only leadership in N.S. also utilized supportive messaging. It was found that a more direct or aggressive approach was also evident, with the premier’s message, “Stay the Blazes Home” [102], becoming a catchphrase during the first wave. In response to people not following the public health orders and adhering to restrictions, the premier stated:

*“I am still hearing stories of people driving to our parks and beaches…these people are the reckless few and not only are Dr. Strang and I upset with them, their fellow Nova Scotians are upset with them. To those reckless few: if you won’t do your part to keep physical distance to help flatten the curve, police will do it for you.”*

[102] (para. 7)

Messaging from leadership plays a critical role in engaging citizens and ensuring that the public takes the situation seriously, supporting and cooperating with the general public [103]. News media indicates that supportive messaging is linked to improved safety in the Atlantic region and the development of grassroots initiatives.

### 4.3. The General Public Embraces the Governmental Decisions Generated by the Dual-Gendered Leadership

The Atlantic region has been lauded for decreasing infections and effective responses (e.g., isolation measures, travel restrictions, and effective contact tracing) [104]. With an understanding of the successes in the Atlantic provinces, a brief analysis of grassroots initiatives further demonstrated the merits of dual-gendered leadership.

#### 4.3.1. Encouraging Grassroots Collaboration

The success of measures in the Atlantic Bubble has been viewed by epidemiologists and public health experts as a template for other provinces across Canada, with N.B. Premier and Dr. Russell proclaiming that the Atlantic provinces were “the envy of Canada” [52] (para. 12). It appears as though a degree of success can be attributed not only to the guidance exhibited by the leadership in the Atlantic province but to the citizens themselves. As Miller and Zafar [104] reflected in their news article:

*“I do feel like the response from the public in the Atlantic region is different than other parts of the country…I think there’s also a certain amount of pride that we have been able to maintain the bubble, and I don’t think that people want to see it change.”*

[104] (para. 10 and 11)

With lower case counts and an acceptance that measures have generally been effective compared to other provinces, it is vital to review grassroots measures to determine if the public truly heeded and adjusted in response to messaging from dual-gendered leadership teams. News media indicates that the messaging was relayed effectively and local communities took the messaging into action, according to the new reality posed by COVID-19.

#### 4.3.2. Supporting People in Need

At the grassroots level, community-based initiatives have been widely covered by the public media, having been undertaken to heed the messages of supporting one another during the pandemic. Grassroots activities exhibit that evidence adaptation has occurred at the local level in response to the pandemic and subsequent measures enacted by provincial leadership.

In particular, with the first cases of the virus in small communities in rural areas of N.L., the communication of the dual-gendered leadership team filtered down to smaller communities; the local mayors communicated amongst one another and responded accordingly while public health contact tracing occurred, rallying rural residents [105]. The onset of COVID-19 exacerbated Indigenous people’s food and income security issues, accelerating the launch of garden initiatives across Atlantic Canada in First Nation communities [106]. During the lockdown in the first wave, community-based service organizations in Saint John, N.B. provided food for “students and families who rely on the school lunch program” [107] (para. 1). The Salvation Army in Port aux Basques, N.L. made meals for essential workers and truckers who worked for the supply and distribution lines for the island, with the spokesman remarking, “this is about making a difference. It’s about meeting a need at such a time as this” [108] (para. 3).

As previously mentioned, dual-gendered leadership has conveyed strong, caring messages for vulnerable and marginalized groups. Local communities converted these messages into action to address the unique needs of these groups, with these cases demonstrating the ripple down effects of dual leadership.

## 5. Discussion

The impact of COVID-19 has had a disproportionate effect on and exposed long-standing economic and social inequities among women [109]. Despite the recognition of the significant impact COVID-19 has had on women, little attention has been paid to gender differences that take place in the process of public health emergency decision making. This is possible because emergency management leadership has been man-dominated [7]. With women occupying senior leadership positions in the Atlantic region, there is an opportunity to explore strategies utilized under dual-gendered leadership concerning public health risk reduction. An analysis of media coverage indicates that this model is effective on several fronts through public health risk mitigation approaches and the responsivity of residents. Indeed, the Atlantic region has not reported significant non-essential international travel among governmental officials compared to other provinces across Canada [110]. Governmental officials’ non-essential travel within the Atlantic Bubble has been limited or exhibited evidence of adherence to public health measures [111]. In addition, the dual-gendered provincial leadership has provided timely responses and explanations to address the general public’s concerns.

### 5.1. Promoting Dual-Gendered Leadership in Disaster and Emergency Management

This scoping media coverage review challenges the notion that protectionism is found in a man-only leadership as it was present in both models. A critical difference between the existing literature and this case study is that dual-gendered leadership exhibits a higher risk aversion than expected from previous literature [26,27]. The complexity of COVID-19 and the public health decision-making process illustrate that further investigation is needed to examine various merits of dual-gendered leadership comprehensively. Moreover, this research has added to the notion that gender diversity in scientific-political communication benefits the general public, specifically in increased awareness and educational measures directed towards the general public. This analysis indicates that supportive language and communication strategies rather than an aggressive approach allow for clear messaging directed towards the general public while supporting COVID-19-driven vulnerable groups. The gender-informed public health communication approach encourages the engagement of citizens to collectively contribute efforts to curb the spread of COVID-19 and stimulates grassroots initiatives that adhere to public health measures while adapting to new realities in very uncertain times.

In addition, the response of provincial governments to public critiques provides further insight into the general public’s view regarding the strengthens of dual-gendered leadership. The COVD-19 pandemic has dramatically reshaped society, triggering catastrophic influences on inhabitants’ and co-inhabitants’ physical health, mental wellness, and overall well-being [112]. The related public health interventions also caused unfavorable or aggressive behaviors of citizens, directly targeting the women CMOs. In fact, at the federal and provincial levels, Dr. Tam (federal), Dr. Bonnie Henry (British Columbia), and Dr. Morrison (P.E.I.) have received abusive or frightening feedback, or “death threats” [48]. Since N.S. CMO (man) has also received the same threats, it is not reasonable to conclude that women leaders have faced more criticism than their men peers [45]. However, in a study that analyzed Twitter data regarding feedback towards public health officers in Canada, men provided “overwhelmingly positive” comments towards Dr. David Williams, the CMO of Ontario, compared to other women CMOs [48]. Consequently, further comparisons between man-only and dual-gendered leadership in Canada could identify the general public’s thoughts towards the leadership in each province’s setting.

### 5.2. Limitations and Mitigation Strategies

Although a scoping media coverage review is an effective approach to include media associated with gender-specific scientific-political communication, the limitations of this study are clear. With regards to data curation, this study only reviewed news articles from the three top-rated media outlets in Canada during the first two waves of COVID-19. This approach potentially excluded news from other public media outlets, which might deepen the analysis. Moreover, this case study focused on English news articles because all four provinces in the Atlantic region use English as their official language [113]. It is worth mentioning that N.B. is a bilingual province; however, French is the minority [114]. As a multilingual country, Canada features a number of residents that do not use either of the official languages (English and French). Excluding the news articles published in French or other languages might neglect some supportive information, especially different possible grassroots responses and adherence towards provincial public health policies and interventions.

Generally, authors’ professional backgrounds have dramatically influenced the data analysis in most qualitative studies [115]. Hence, the two authors reviewed all the news articles independently and developed themes to reduce their personal bias in data interpretation and article conceptualization. Furthermore, the media’s potentially inherent political and social preferences, further impacting the general public’s interpretation of governmental interventions, cannot be avoided entirely within media outlets. It is difficult to identify the specific bias without compromising the material within articles and news stories. Accordingly, this study balanced the number of new articles among the three media companies in order to merge various standpoints. The authors also caution regarding the generalizability of this review to other provinces across Canada that also feature dual-gendered leadership (e.g., British Columbia, Alberta, and Ontario) due to societal differences (e.g., population, cultural, and economic) among them and what are found within Atlantic Canada.

Furthermore, media coverage generally illustrates the final stage of governmental interventions and briefly notices the decision-making process. However, the overall decision-making process at the government level engages various departments and related stakeholders. It is understandable that institutional power and related power dynamics among the different provincial government departments dramatically inform the governmental operation and final policies [116]. However, the institutional tensions might not be directly identified through the media coverage review. Hence, it might not be easy to further examine the gender role in the institutional strains. Future studies might consider other data collection approaches (e.g., governmental officials’ interviews and governmental documents analysis) to provide a comprehensive understanding of the influences of dual-gendered leadership.

Moreover, although Atlantic provinces share some similarities with one another in the Atlantic region and other provinces and territories across Canada, their tremendous differences at the regional and national levels indicate that the outcomes of these studies might not be directly mobilized to different contexts. The limitations are apparent; relative mitigation strategies developed above contribute to the trustworthiness of this case study of the Atlantic region in Canada [117], providing a valuable region-based reference regarding advocating for dual-gendered leadership in the governmental intervention for the current public health emergency in particular, and for potential disasters (including public health crises) in general.

## 6. Conclusions

Utilizing a scoping media coverage review approach, this case study of Atlantic Canada provides a glimpse into dual-gendered leadership and associated measures that may play a role in mitigating risk in response to the first two waves of COVID-19. In the Atlantic Bubble, women CMOs have influenced the scientific-political communication and improved political interventions during the first and second waves of the pandemic in Atlantic Canada through closely collaborating with their men counterparts and effectively communicating with the general public. Specifically, at the public health policymaking level, the women CMOs prioritized the health and well-being of citizens over economic interests through a more balanced approach to reopening once public health risks were deemed to be mitigated. Their leadership roles promoted advocacy in messaging related to vulnerable and stigmatized populations and facilitated cross-departmental cooperation at the provincial government level. These contributions have enhanced the governmental communication strategies to deliver consistent, supportive, and concise messaging while dispelling misinformation and inconsistent communication, continually building trust and confidence with the general public. The measures taken within a dual-gendered leadership approach indicate that with increased trust and engagement through communication with the general public, grassroots initiatives not only adhere to public health protocols but adapt to a changing landscape brought on by the COVID-19 pandemic in Atlantic Canada.

This case study focuses on the dual-gendered leadership in the first and second waves of COVID-19 in the Atlantic Bubble. Despite the successes, caution should be given about generalizing and mobilizing the benefits of dual-gendered leadership to potential waves of COVID-19, other extreme events (including public health crises), and domestic and international communities. Currently, although dual-gendered leadership successfully guided Atlantic Canada through the third wave of COVID-19 [118], a longitudinal and retrospective study with all the waves (past, current, and future) could further evaluate dual-gendered leadership strengths and weaknesses over time. Since this dual-gendered leadership is also utilized in other provinces, a Canada-wide comparison that considers societal differences will contribute to a nuanced understanding of dual-gendered leadership. If possible, a cross-national comparison including international communities utilizing a similar dual-gendered leadership approach will provide knowledge of international efforts to respond to the COVID-19 pandemic.

## Figures and Tables

**Table 1:** Premiers and chief medical officers (CMOs) in Atlantic Canada.

Atlantic Province	Premier	Chief Medical Officer	Leadership Type
New Brunswick (N.B.)	Mr. Blaine Higgs	Dr. Jennifer Russell	Dual-gender
Nova Scotia (N.S.)	Mr. Stephen McNeil	Dr. Robert Strang	Man-only
Prince Edward Island (P.E.I.)	Mr. Dennis King	Dr. Heather Morrison	Dual-gender
Newfoundland and Labrador (N.L.)	Mr. Dwight Ball Mr. Andrew Furey *	Dr. Janice Fitzgerald	Dual-gender

* The new premier assumed the role on 19 August 2020 [18].

**Table 2:** The numbers of initial search and final reviewed news articles.

Province/Country	CBC News		CTV News		The Globe and Mail		Sub-Total	
	Initial Search	Final Reviewed	Initial Search	Final Reviewed	Initial Search	Final Reviewed	Initial Search	Final Reviewed
Canada	419	2	185	0	226	15	830	17
N.B.	345	14	141	0	31	6	517	20
N.S.	247	2	253	10	29	8	529	20
P.E.I.	337	9	66	0	14	5	417	14
N.L.	227	9	23	0	15	3	265	12
Total	1575	36	668	10	315	37	2558	83

**Table 3:** Themes, sub-categories, and codes.

Themes	Sub-Categories	Codes
Influences on the provincial government public health decision making	Mitigation Role	Economic impacts, Social impacts, Health impacts, A scientific-driven approach, risk mitigation
	Advocating Role	Vulnerable people, Stigmatized populations
	Coordination Role	Cross-Departmental Collaboration, Cross-party collaboration, Cross-provincial Collaboration
Influences on dissemination of public health Information	Information Delivery	Consistent Messages, Avoiding Misdirecting, Timely clarification, Personal messages
	Build Trust	Supportive and encouraging messages, Customized messages, Direct and aggressive approach, Self-care
Influences on local communities	Encouraging Grassroots Collaboration	Maintain the bubble, Community effects, Support public health protocols
	Supporting People in Need	Urban residents’ activities, Rural residents’ activities, Addressing urgent needs, Community service agency efforts

## Data Availability

All data used to develop this article are available in a repository online DesignSafe-CI (DOI: 10.17603/ds2-g757-2079) in accordance with funder data retention policies.
